# Spontaneous and strong multi-layer graphene n-doping on soda-lime glass and its application in graphene-semiconductor junctions

**DOI:** 10.1038/srep21070

**Published:** 2016-02-12

**Authors:** D. M. N. M. Dissanayake, A. Ashraf, D. Dwyer, K. Kisslinger, L. Zhang, Y. Pang, H. Efstathiadis, M. D. Eisaman

**Affiliations:** 1 Sustainable Energy Technologies Department, Brookhaven National Laboratory, Upton, NY 11973, USA; 2 Department of Physics and Astronomy, Stony Brook University, Stony Brook, NY 11794, USA; 3 Colleges of Nanoscale Science and Engineering (CNSE) at SUNY Polytechnic Institute, Albany, NY 12203, USA; 4 Center for Functional Nanomaterials, Brookhaven National Laboratory, Upton, NY 11973, USA; 5 Department of Electrical & Computer Engineering, Stony Brook University, Stony Brook, NY 11794, USA.

## Abstract

Scalable and low-cost doping of graphene could improve technologies in a wide range of fields such as microelectronics, optoelectronics, and energy storage. While achieving strong p-doping is relatively straightforward, non-electrostatic approaches to n-dope graphene, such as chemical doping, have yielded electron densities of 9.5 × 10^12^ *e*/cm^2^ or below. Furthermore, chemical doping is susceptible to degradation and can adversely affect intrinsic graphene’s properties. Here we demonstrate strong (1.33 × 10^13^ *e*/cm^2^), robust, and spontaneous graphene n-doping on a soda-lime-glass substrate via surface-transfer doping from Na without any external chemical, high-temperature, or vacuum processes. Remarkably, the n-doping reaches 2.11 × 10^13^ *e*/cm^2^ when graphene is transferred onto a p-type copper indium gallium diselenide (CIGS) semiconductor that itself has been deposited onto soda-lime-glass, via surface-transfer doping from Na atoms that diffuse to the CIGS surface. Using this effect, we demonstrate an n-graphene/p-semiconductor Schottky junction with ideality factor of 1.21 and strong photo-response. The ability to achieve strong and persistent graphene n-doping on low-cost, industry-standard materials paves the way toward an entirely new class of graphene-based devices such as photodetectors, photovoltaics, sensors, batteries, and supercapacitors.

The benefit of using of chemical-vapor-deposited (CVD) graphene as a passive transparent electrode^1^,^2^ is well recognized, but its potential to be paired with a semiconductor and play an active role as part of an electronic junction remains a very active field of research^3^,^4^,^5^,^6^,^7^. To enable more control in fabricating active graphene-semiconductor junctions, pristine CVD graphene must be doped p-type or n-type, since unlike epitaxial graphene^8^, it is not doped upon growth. Electrostatic^4^,^5^,^6^ and chemical doping^7^ have resulted in Schottky diodes between p-doped graphene and n-type silicon. However, unlike p-doping, which occurs even naturally for graphene exposed to atmospheric water molecules^9^, persistent graphene n-doping with high electron density that is resistant to degradation has been more difficult to achieve. To this end, nitrogen based precursors during growth^10^ as well as amines^11^,^12^ and transition/alkali metals^13^,^14^,^15^,^16^,^17^ after growth have been explored. Although these approaches have shown promise in highly controlled experimental settings, all existing persistent n-doping techniques^10^,^11^,^12^,^13^,^14^,^15^,^16^,^17^ fail to achieve the strength (more than 9.5 × 10^12^ *e*/cm^2^ (ref. 11)), robustness, and scalability ultimately required for most applications^1^,^2^. Previous reports have shown that adsorbed alkali-metal atoms cause strong n-doping in graphene, but challenges remain, such as the reactivity of alkali metals and the lack of a scalable process^15^. We demonstrate that an alkali metal (Na) embedded in inert, industrial-grade (~8% Na_2_O), low-cost soda-lime glass (SLG) overcomes these challenges and strongly n-dopes graphene (1.33 × 10^13^ *e*/cm^2^, see Fig. S1 in the Supplementary Information for comparison to the n-doping strength achieved in refs 10, 11, 12, 13, 14, 15) via surface-transfer doping from Na, upon transfer of CVD-grown graphene onto the SLG. Initial tests show that the doping strength does not degrade when the devices are left in air for several weeks, which is already superior to previously demonstrated methods. The persistence of the doping achieved with this technique (that is, the resistance to degradation) is due to the effectively inexhaustible reservoir of Na in the SLG, in contrast to chemical doping where the doping strength degrades due to the reactivity or evaporation of the finite amount of externally introduced dopants^10^,^11^,^12^.

A field-effect transistor (FET) with multi-layer graphene (GR) transferred onto SLG (8% Na_2_O) was characterized to obtain a plot of conductance (G) (maximum normalized to one) vs. gate-voltage (V_G_) (Fig. 1A). The minimum G value (i.e., Dirac point) is seen at −67 V, indicating n-doping of 1.33 × 10^13^ *e*/cm^−2^ and a Fermi energy shift (ΔE_F_) of +426 meV. Photoelectron spectroscopy and secondary ion mass spectrometry (SIMS) of Na in the SLG (supplementary Figs 4 and 5) indicate a Na surface density (ρ_Na_) of ρ_Na_ = 1.15 ± 0.05 × 10^14^ cm^−2^ at the surface adjacent to graphene; similar densities of alkali metals on graphene are known to induce strong n-doping via electron transfer from the metal atoms to graphene^16^,^17^. Density functional theory (DFT) calculations (Supplementary section 10) that assume a Na monolayer on graphene (with ρ_Na_ = 7.6 × 10^14^ cm^−2^–close to the measured surface-density of Na on SLG), show n-doping with ΔE_F_ = + 474 meV (Fig. 1B), in close agreement with the experimental measurements (ΔE_F_ = + 426 meV). The measured charge-transfer rate of 0.11*e* per-Na-atom is also in agreement with literature values of 0.1*e*–0.2*e*^13^,^17^. As a control experiment, the transconductance of a graphene FET on a low-Na (<1% Na_2_O) borosilicate-glass (BSG) substrate shows a Dirac point at V_G_ ~0 V (inset Fig. 1A), indicating no doping and attributable to the lower ρ_Na_ (~2.30 ± 0.3 × 10^13^ cm^−2^, Fig. S5) of BSG compared to SLG. These measurements confirm that the strong graphene n-doping on SLG is caused by charge transfer from the high density of Na near the surface.

Next, to test if graphene/p-type semiconductor/SLG substrates result in n-doping of graphene via Na diffusion through the semiconductor, and the corresponding formation of a p-n junction, we deposited p-type CIGS onto the Mo-side of a Na-rich SLG substrate coated with 330 nm of Mo, and then transferred graphene onto the CIGS surface. CIGS/Mo/SLG was chosen because it is a standard substrate used in the solar industry, and so our measurements will help determine the feasibility and versatility of this doping method in real-world applications. SLG substrates are commonly used because Na in CIGS is known to improve solar cell performance^18^,^19^. During co-evaporation of CIGS on to Mo/SLG above 550 °C (evaporated Mo layer on SLG serves as a contact), Na diffuses from the SLG into the CIGS along the grain-boundaries seeking oxygen for an octahedral co-ordination^20^, ultimately forming Na_2_CO_3,_ NaSeO_3_, or NaOH at the CIGS/air surface^18^,^21^. Figure 1C indicates that the Na concentration in CIGS increases rapidly near the CIGS/air surface, reaching a value of ρ_Na_ = 4.18 × 10^13^ cm^−2^ at the surface. In contrast, the CIGS/air surface ρ_Na_ in CIGS/Mo/BSG is nearly two orders of magnitude lower (ρ_Na_ = 6.16 × 10^11^ cm^−2^).

The HR-TEM in Fig. 1D shows that the graphene on top of the CIGS/Mo/SLG substrate is multi-layer graphene with 5-layers and an interplanar spacing of 340 pm, in agreement with previous reports for pristine graphene (335 pm)^22^. Raman spectroscopy (Fig. 1E) shows the primary CIGS peak^23^ at 177 cm^−1^, along with the graphene G peak at 1585 cm^−1^ and the 2D peak^24^ at 2665 cm^−1^; the graphene D peak^24^ at 1350 cm^−1^ is negligible, indicating minimal damage during transfer to the rough CIGS surface (Fig. S2). Energy dispersive spectroscopy (EDS) (Fig. 1F) reveals that Na is distributed uniformly (top-panel) in-plane along the GR/CIGS interface, making electronic interactions between the Na and graphene extremely likely (Fig. 1G). We note that by using a configuration in which the Na host (not necessarily SLG) is in direct contact with graphene (Na-host/graphene/semiconductor), this approach can be used to form n-graphene/p-semiconductor junctions for a wide range of p-type semiconductors.

The doping effects and junction properties of graphene on CIGS/Mo/SLG (and on CIGS/Mo/BSG) are investigated using the four-terminal device in Fig. 2A. Figure 2B shows an SEM top-view and the inset shows a TEM cross-section. Figure 2C shows the conductance (normalized) vs.V_G_ and the ΔE_F_ of each device (schematic on right). For GR/CIGS/Mo/SLG (red-curve), the conductance minimum V_G_ (Dirac point) exceeds the measurement limit and is estimated via extrapolation to be V_G_ = −106 V (Fig. S8), confirming strong n-doping (2.11 × 10^13^ *e*/cm^−2^) with ΔE_F_ = +536 meV, surpassing the ΔE_F_ for graphene on neat SLG (green-curve). This strong n-doping results from the high Na density that diffuses through CIGS and concentrates at the CIGS/air surface (ρ_Na_ = 4.18 × 10^13^ cm^−2^, Fig. S7). This conclusion is supported by the fact that graphene on CIGS/Mo/BSG (yellow-curve) with CIGS/air surface ρ_Na_ = 6.16 × 10^11^ cm^−2^ (Fig. S7) is not doped, similar to GR/BSG (cyan-curve). Furthermore, ΔE_F_ = + 536 meV of GR/CIGS/Mo/SLG agrees with the potential-shift (ΔΦ) (+698 meV) due to the Na-ion dipole interaction on graphene, as calculated using Helmholtz equation (see Supplementary Information, Section 6)^25^. Comparatively, the calculated shift for GR/CIGS/Mo/BSG with ρ_Na_ = 6.16 × 10^11^ cm^−2^ is ΔΦ ~10 meV, explaining the lack of n-doping. Interestingly, the electron transfer rate from Na to graphene in GR/CIGS/Mo/SLG (0.50*e* per-Na-atom), is higher than in GR/SLG (0.11*e* per-Na-atom), which we attribute to ionization-rate differences of the Na species on the CIGS and SLG surfaces.

Figure 2D is the current-voltage (I–V) curve between graphene-Mo contacts in the dark (dashed), and 11.14 mW cm^−2^ white light (solid), with (red) and without (blue) the Al_2_O_3_ gate-dielectric (Fig. 2A). These measurements show non-linear diode-behavior with a photocurrent response, with much higher performance when the Al_2_O_3_ top layer is present. Relative to the measurements without the Al_2_O_3_ top-layer, the reverse saturation current density (J_0_) with Al_2_O_3_ is reduced by 10^4^ to 0.36 nA/cm^2^ (Fig. S10), the photocurrent is enhanced by a factor of 650 at −10 mV, and the ideality factor becomes as low as 1.29. Figure 2C shows that a control GR/SiO_2_/p-Si FET without the Al_2_O_3_ dielectric (dashed blue curve) has a Dirac point beyond V_G_ = +100 V, indicating strong p-doping; a GR/SiO_2_/p-Si FET with the Al_2_O_3_ dielectric (Fig. 2C, solid blue curve) shows much less p-doping, with a Dirac point near V_G_ ~ 20 V. These measurements demonstrate that the Al_2_O_3_ dielectric shields the graphene from p-dopants in ambient air, resulting in an enhanced Schottky barrier (Φ_b_) and built-in field.

Figure 2E shows I–V curves of GR/CIGS/Mo/SLG at different V_G_, where J_0_ is reduced as V_G_ is increased from −100 V to 50 V. Plotting ln(*J*_0_) vs. (|*V*_*G*_ + Δ*V*_*F*_|)^1/2^ , we obtain Φ_b_ = 0.29 eV (Supplementary Information, Section 8). The photocurrent at zero-bias increases as graphene is n-doped further by increasingly positive V_G_ (Fig. 2F), which we attribute to the concomitant increase in the built-in field. Moreover, under 1000 W m^−2^ illumination at V_G_ = 0, the photocurrent is 13.6 mA/cm^2^, yielding a power conversion efficiency of ~1%; this represents the first demonstration of n-graphene/p-CIGS photovoltaic behavior. The lowest ideality factor obtained was 1.21 (Fig. S11), indicating negligible recombination in the space-charge region^26^.

The behavior of J_0_ vs. temperature (T) is modeled assuming Landauer transport^5^ in the GR/CIGS/Mo/SLG (Fig. 3A) giving Φ_b_ = 0.13 eV. Assuming ideal Schottky-diode behavior, ln(*J*_0_/*T*^2^) vs. *1/T* (inset to Fig. 3A) yields Φ_b_ = 0.11 eV, while ln(*J*_0_) vs. (|*V*_*G*_ + Δ*V*_*F*_|)^1/2^ (Fig. S13) yields Φ_b_ = 0.29 eV at zero gate bias, with a constant Richardson coefficient of 1.18 × 10^−6^ mAcm^−2^K^−2^. As is discussed below, this range for Φ_b_ (0.11 eV–0.29 eV) is lower than expected, which we believe is due to surface defects and surface sodium doping of CIGS that lowers the surface ionization potential of CIGS relative to the bulk.

The barrier height (Φ_b_) is equal to the ionization potential of the CIGS semiconductor (IP_CIGS_) minus the work function of graphene (Φ_G_), and represents the barrier that a hole in the valence band of CIGS must overcome to reach the graphene interface and recombine with an electron there. The activation energy (E_a_), on the other hand, represents the characteristic energy that governs the rate of minority carrier (electron) excitation into the conduction band of the p-type semiconductor CIGS. In an intrinsic semiconductor, E_a_ is equal to half the bandgap, and for a doped p-type semiconductor like CIGS, its value should be close to the bandgap energy, since in this case the Fermi level is close to the valence band.

While fitting the *J*_*0*_ *vs. T* data to the Schottky barrier model allows us to determine Φ_b_, fitting this same data to the modified-Arrhenius/activation-energy model (Eqs (S11 and S12) and Fig. 3B) allows us to determine a value for *E*_*a*_, which will indicate whether the recombination is predominantly bulk or interfacial. The ideality factor *n* is expected to become temperature dependent in the presence of tunneling. The measured I–V data is used to determine *n* as a function of temperature and this is directly incorporated into Eq. (S11), which allows us to separate the effects of *E*_*a*_ and *n* on the reverse saturation current density *J*_*0*_. Figure 3B shows a modified Arrhenius-plot of *n*ln(*J*_0_) vs. *1/T* (where *n* is the ideality factor), yielding an activation energy (E_a_) of 0.96 eV, which indicates dominant interfacial recombination since it is less than the CIGS bandgap of 1.15 eV (Ref. 26). We have demonstrated that this interfacial recombination can be reduced using a very thin (4 nm) TiO_2_ blocking layer between graphene and CIGS, thereby improving V_oc_ from 0.23 V to 0.49 V (Fig. S14). The space-charge width (W_d_) of the diode is measured to be 190 nm using C-V measurements (Fig. 3C). The approximate band structure of the Schottky diode is given in Fig. 3D. The difference between the CIGS ionization-potential (IP_CIGS_ = 5.65 eV (ref. 27)), and graphene work function 4.69 eV (ref. 28) modified by the image-potential correction (0.15 eV), gives a theoretical Φ_b_^T^ = 0.81 eV. Due to defects^29^ and Na surface density^30^, IP_CIGS_ is ~0.5 eV lower, yielding Φ_b_^T^ = 0.31 eV, which is much closer to the measured range of Φ_b_ = 0.11 eV–0.29 eV. It is worth noting that even though the best-fit range of Φ_b_ (0.11 eV–0.29 eV) is much less than the best-fit value of *E*_*a*_ (0.96 eV), both models yield good fits to the same *J*_*0*_*vs. T* data due to the inclusion of the temperature-dependent *n* in the activation-energy model.

In conclusion, we have demonstrated strong (1.33 × 10^13^ *e*/cm^−2^, corresponding to a Fermi energy shift of +426 meV.), robust, and spontaneous n-doping of graphene on the surface of a low-cost industrial-grade soda-lime-glass substrate via surface-transfer doping from the Na. By leveraging the Na diffusion through a p-type CIGS semiconductor deposited onto the soda-lime glass, we applied this method to the formation of a graphene(n)/semiconductor(p) Schottky diode with even stronger graphene n-doping (2.11 × 10^13^ *e*/cm^−2^, corresponding to a Fermi energy shift of +536 meV) than was achieved on bare glass. This method of n-doping does not require any high-temperature annealing steps, and should be compatible with a wide range of semiconductor/substrate systems. The junction properties, such as Schottky barrier height and interfacial recombination rate, can be controlled by tuning the doping strength via the thickness of a few-nm dielectric layer such as TiO_2_ or Al_2_O_3_. Advantages of this technique include the lack of external chemicals whose doping strength decays over time, the ability to achieve strong and persistent n-doping of graphene that is placed on top of a p-doped semiconductor, the ability to n-dope graphene on a wide range of p-doped semiconductors via the use of a Na host that is in direct contact with the graphene layer, and the ability to control the strength of the doping via the use of a spacer layer (e.g., TiO_2_) between the Na host and the graphene layer. Disadvantages include the possible restriction to p-doped semiconductors that are not too strongly affected by the Na diffusion from the Na host to the graphene layer, in the case where the semiconductor lies between the Na host and the graphene layer.

Strong, robust, and tunable graphene doping opens the door for the practical realization of many envisioned applications of graphene such as touch screens and organic light-emitting diodes^1^, where the reduction of sheet resistance is crucial to future success, and a broad array of other applications where strong and tunable n-doping is important, such as microelectronics, photodetectors, photovoltaics, electrochemical energy storage, and sensors^2^.

## Methods

### CIGS deposition on Mo/SLG

Given in Supplementary Information, Section 2.

### Device Fabrication

Supplementary Fig. S3 shows a schematic of the graphene/CIGS device fabrication process. In order to make our GR/CIGS devices, 450 nm of SiO_2_ is first deposited on top of the CIGS/Mo/SLG (BSG) substrates via plasma-enhanced chemical vapor deposition (PECVD) at 160 °C at 1.6 nm/s rate. Next, 1 × 1 μm^2^–500 × 500 μm^2^ regions were patterned on the PECVD SiO_2_ either using optical lithography (or ebeam lithography) techniques depending on the feature size. E-beam was performed using the E-beam lithography JEOL JBX-6300FS system on E-beam resist positive resist ZEP520A (spun at 2000 rpm for 40 sec annealed at 180 °C for 3 minutes) by exposing with a dose of 400 μC/cm^2^ at 100 keV and developed with hexylacetate for 90 sec. In optical lithography, the tool MA6 Mask aligner was used with positive optical resist S1811spun at 4000 rpm for 45 sec annealed at 110 °C for 1 minute, and developed with MIF 312 3:2 with DI water for 1 minute. These patterned regions were then etched via Reactive Ion Etching (RIE) (Oxford Plasmalab 100 ICP etcher) using a mixture of (CHF_3_ and Ar) at 15 nm/min, until the CIGS was exposed. Commercially obtained CVD graphene on Cu foil (Graphene Platform) was then transferred from the Cu substrates to the SiO_2_/CIGS/Mo glass substrates. The graphene transfer was done by coating the graphene side of the graphene/Cu foils with PMMA (10% w/w in chlorobenzene spun at 3000 rpm for 1 min sec and annealed at 140 °C per 1 min), oxygen plasma etching (March Plasma Etcher, 20 W, 100 mT for 20 sec) the opposite side, and etching the Cu using ammonium persulfate (0.1 M) solution overnight. As the Cu is etched away, the graphene/PMMA film floats on the etchant and it is washed in de-ionized water (>18 MΩ resistivity using a Millipore DI system) and it then transferred, graphene-side down, onto the pattered CIGS/Mo/SLG or other control substrates such as neat SLG or BSG substrate. Afterwards, graphene transferred substrates are annealed at 100 °C for 30 minutes in a vacuum oven to remove water, and are subsequently annealed at 200 °C for 15 minutes to soften the PMMA and promote conformal adhesion onto substrates. It is found that the 200 °C annealing step is extremely critical in getting highly uniform, wrinkle- and damage-free graphene films on the rough surfaces of CIGS and SiO_2_ substrates. Afterwards, the PMMA is removed from the graphene by immersing in acetone overnight and the substrate is further annealed in a Rapid Thermal Annealer at 375 °C in Ar (96%): H_2_(4%) forming gas for 15 minutes for complete PMMA removal. Next, the graphene is etched following optical (ebeam) lithographic patterning using oxygen plasma etch. (March plasma, 100 W and 100 mT for 1 minutes or Oxford Plasma Lab DRIE at 20 °C for 20 seconds in O_2_) using a negative tone resist mask (E-beam lithography uses ma-N 2403, spun at 2000 rpm for 30 sec exposed at 200 μC/cm^2^ dose for 100 keV for electron beam and developed using ma-D 532 negative tone developer for 1 minute, Optical lithography uses maN-1410 negative resist spun at 3000 rpm for 30 seconds exposed and developed in ma-D 533 for 1 minute). After etching the graphene, source-drain electrical contacts (Au (30 nm)/Cr (5 nm)) are deposited using ebeam evaporation after optical (ebeam) lithography patterning. Next, a 200 nm top gate-dielectric layer (Al_2_O_3_) is blanket deposited on GR/CIGS/Mo/SLG(BSG) or GR/SLG(BSG) substrates via Atomic Layer Deposition at 1 Ǻ/cycle using (Tri Methyl Aluminum) TMA/Water precursor at 250 °C. On top of the Al_2_O_3_, a semi-transparent top-gate (10 nm of Au) is deposited via ebeam evaporation following optical (ebeam) lithography patterning. Next, the source and drain electrodes are exposed through the dielectric layer by RIE etching of Al_2_O_3_ using BCl_3_ by Oxford Plasmalab 100 ICP etcher, on a mask pattern using optical (ebeam) lithography.

### Characterization

Given in Supplementary Information, Section 3.

## Additional Information

**How to cite this article**: Dissanayake, D. M. N. M. *et al.* Spontaneous and strong multi-layer graphene n-doping on soda-lime glass and its application in graphene-semiconductor junctions. *Sci. Rep.* **6**, 21070; doi: 10.1038/srep21070 (2016).

## Supplementary Material

Supplementary Information

## Figures and Tables

**Figure 1:**
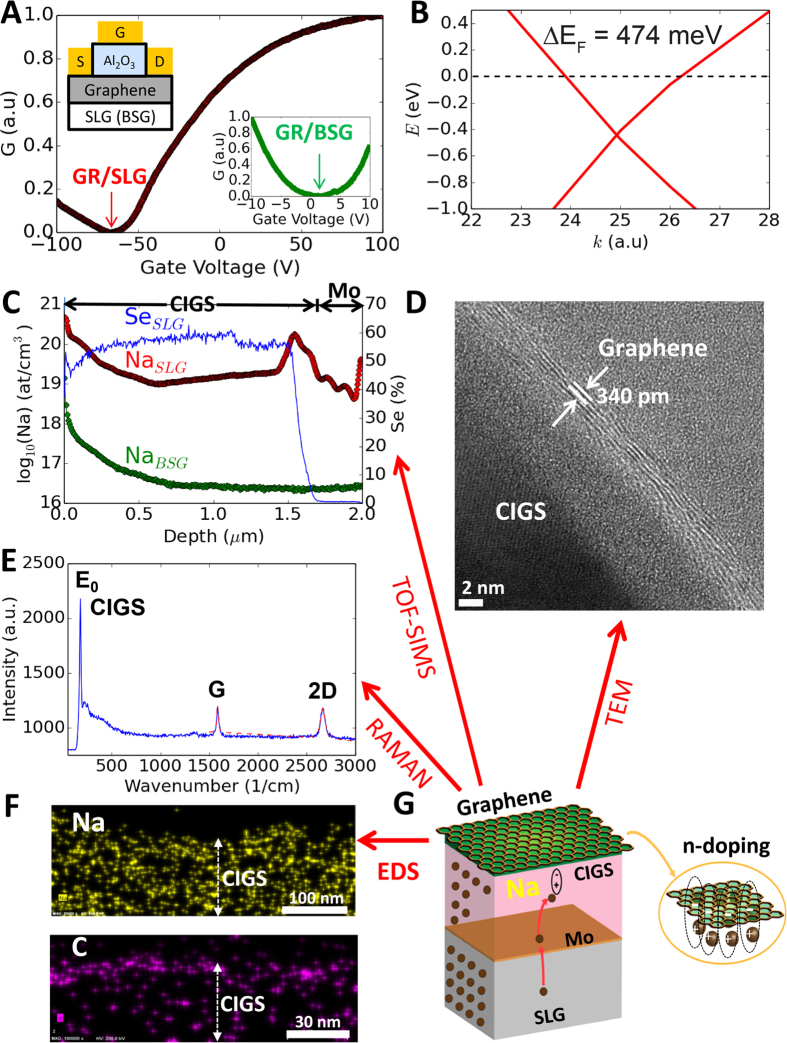
Surface-transfer n-doping of graphene from Na. (**A**) Conductance (G) (normalized) vs. gate-voltage (V_G_) of graphene (GR)/soda-lime glass (SLG) and GR/borosilicate-glass (BSG) (inset) measured in FET configuration (schematic). (**B**) DFT calculated dispersion curve showing n-doping in graphene interacting with Na. (**C**) Na and Se depth-profiles in CIGS/Mo/SLG and CIGS/Mo/BSG from TOF-SIMS. (**D**) Cross-sectional HR-TEM of GR/CIGS/Mo/SLG. (**E**) Raman spectrograph of GR/CIGS/Mo/SLG showing E_0_ peak of CuIn_0_._7_Ga_0.3_Se_2_ (177 cm^−1^), and G peak (1585 cm^−1^) and 2D peak (2665 cm^−1^) of graphene. (**F**) EDS maps of GR/CIGS/Mo/SLG showing Na (yellow, top) and C (purple, bottom). (**G**) Schematic of graphene n-doping mechanism on CIGS.

**Figure 2:**
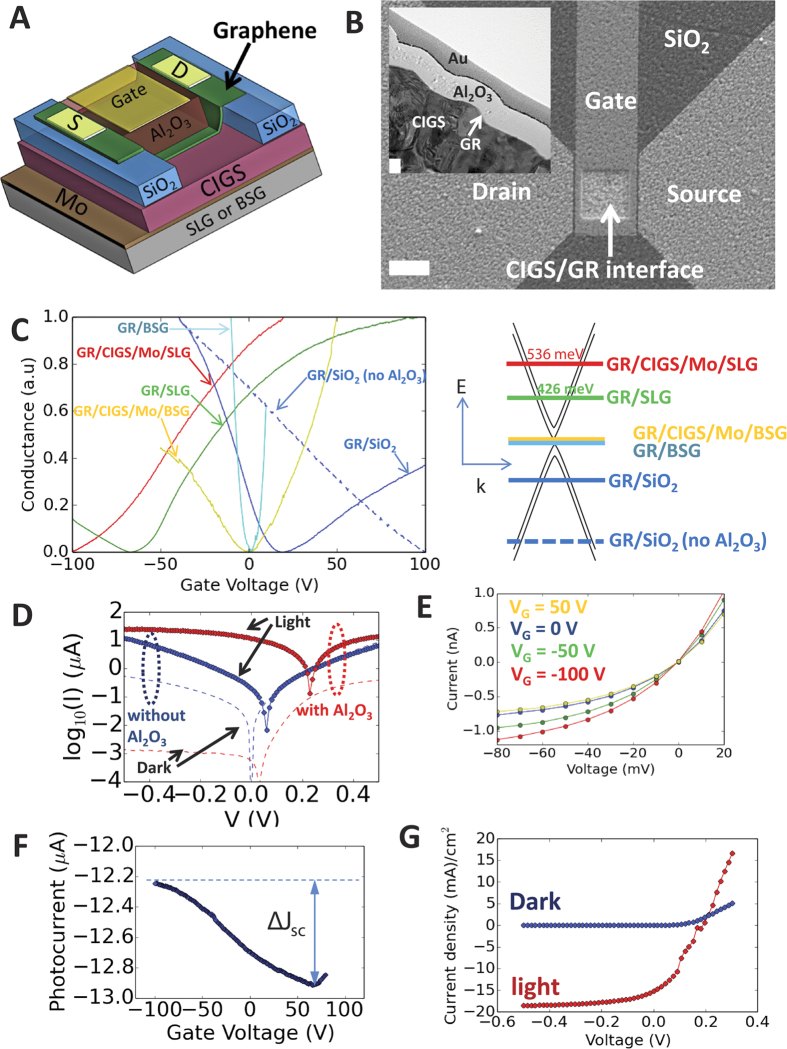
N-doped graphene-CIGS junction. (**A**) Four-terminal GR/CIGS/Mo/(SLG/BSG) FET. (**B**) SEM of device in panel (**A**). Scale-bar is 10 μm. (Inset) TEM cross section. Scale-bar is 100 nm. (**C**) Left: G (normalized) vs. V_G_ in the dark. Right: Band structure for multi-layer graphene with Fermi-level for each sample in plot to left. (**D**) Graphene (source)-Mo (drain) current-voltage (I–V) curve with(red)/without(blue) the Al_2_O_3_ top-dielectric under light(solid)/dark(dotted) for GR/CIGS/Mo/SLG. (**E**) Graphene-Mo I–V at different V_G_ for GR/CIGS/Mo/SLG. (**F**) Photocurrent with V_G_ = 0 V bias under 11.14 mW cm^−2^ illumination. (**G**) I–V of the GR/CIGS/Mo/SLG under 1000 W/m^2^ illumination.

**Figure 3:**
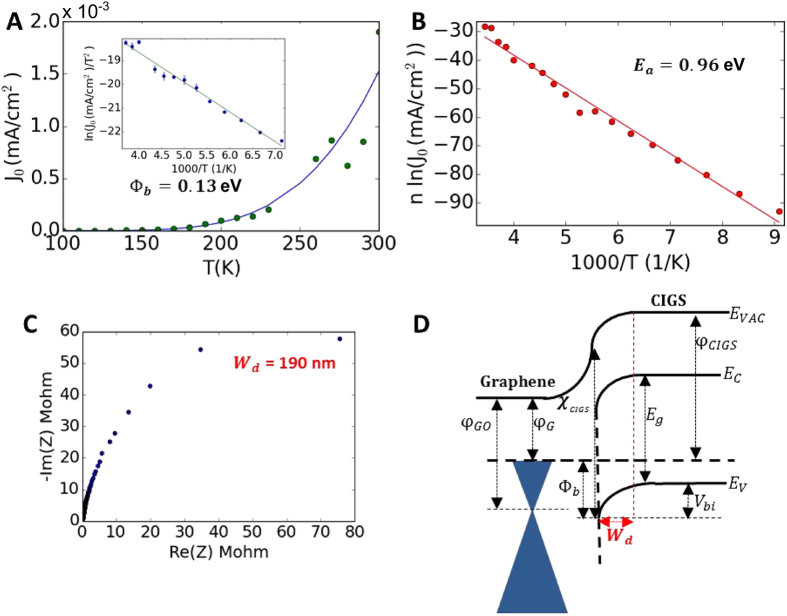
Graphene-CIGS junction properties. (**A**) Data (green circles) and best-fit model prediction (solid blue line) for *J*_*0*_ (mA/cm^2^) vs. *T* for GR/CIGS/Mo/SLG, using a Landauer transport model (see Eq.(S10)) giving Φ_b_ = 0.13 eV. (Inset) Same data (blue circles) plotted as ln(*J*_0_/*T*^2^) vs. 1000/T where *J*_*0*_ is in mA/cm^2^ and T is in K, but using an ideal Schottky diode model, 
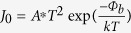
, for the solid green line with best-fit value Φ_b_ = 0.11 eV. (**B**) Same data (red circles) used in panel (a), but plotted as a modified Arrhenius-plot (n*ln(*J*_0_) vs. 1000/T), where *n* is the ideality factor and *J*_*0*_ is in mA/cm^2^; finding the best-fit of Eq. (S12) (solid red line) to the data gives E_a_ = 0.96 eV. (**C**), Nyquist plot from C-V giving depletion width (W_d_) of 190 nm. (**D**) Schematic band structure of multi-layer-GR/CIGS/Mo/SLG interface. φ_G0_ = Work function of intrinsic graphene, φ_G_ = Work function of graphene, Φ_b_ = Schottky barrier height, φ_CIGS_ = Work function of CIGS, IP_CIGS_ = Ionization potential of CIGS, E_G_ = Band gap of CIGS, V_bi_ = Built-in potential.
