# Supplementary material for: Spontaneous and strong multi-layer graphene n-doping on soda-lime glass and its application in graphene-semiconductor junctions
Source: Sci Rep. 2016 Feb 12;6:21070. doi: 10.1038/srep21070 (PMC4751575; doi:10.1038/srep21070)
Supplement: Supplementary Information [file srep21070-s1.pdf]

Supplementary Information

Spontaneous and strong multi-layer graphene n-doping on soda-lime glass and its application in graphene-semiconductor junctions

D. M. N. M. Dissanayake, A. Ashraf , D. Dwyer, K. Kisslinger, L. Zhang, Y. Pang, H. Efstathiadis, and M. D. Eisaman

Correspondence to: meisaman@bnl.gov and nanditha.dissanayake@gmail.com

This PDF file includes:

Supplementary Text
Figs. S1 to S19
Table ST1

1) Doping density comparison

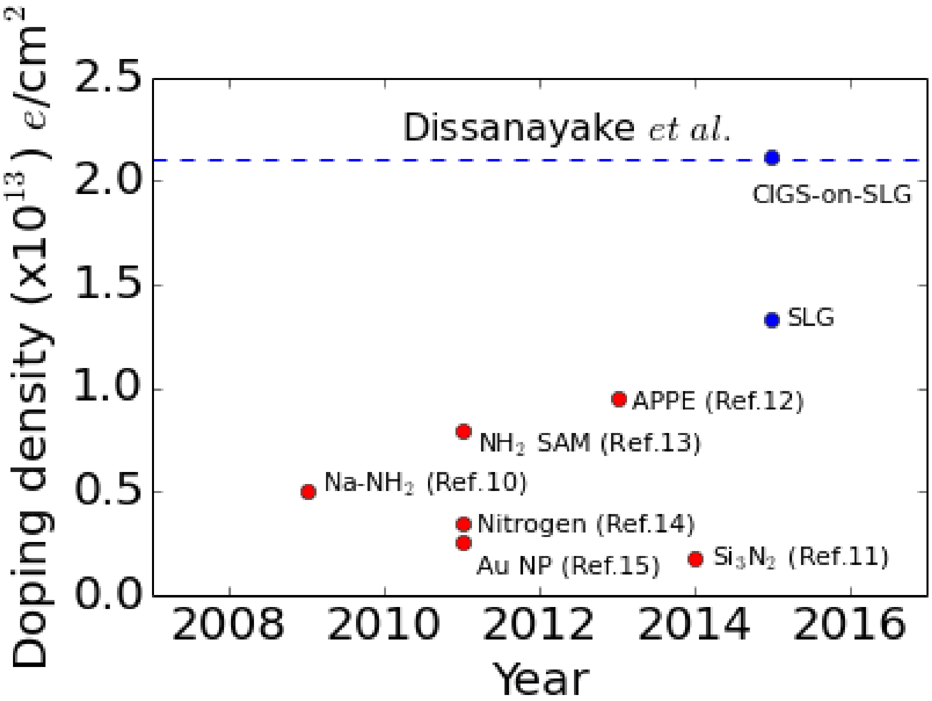

Figure S1. Comparison of n-doping density. Past chemical-doping strengths (red dots) compared to the strength of n-doping achieved in this work (blue dots) via Na surface-transfer doping from soda-lime glass (SLG) and CIGS-on-SLG. APPE = Aminophenyl propargyl ether.

2) CIGS deposition on Mo/SLG

The CIGS films used in these devices were deposited using three-stage thermal co-evaporation from elemental sources of Cu, In, Ga, and Se onto a heated Mo-coated soda-lime glass substrate (SLG). The SLG used was purchased from Guardian (product name: Ecoguard Mo-1, 3.0mm thickness) consisting of nominally ~15% Na_2O (Ref. S1). The glass was coated with a Mo film by the glass manufacturer to a thickness of ~330nm (resistivity 15-22 µΩ-cm). The combined CIGS/Mo film thickness is typically measured to be 2.0µm, with Cu ratio (Cu/(In+Ga)) of 0.94, and Ga ratio (Ga/(Ga+In)) of 0.30 as measured by X-ray Fluorescence (XRF) (e.g. Sample # I1291 shown in Figure S1). XRF is used to measure copper and gallium ratios using a Ceres Technologies System SMX (Model #C06-01915-4002). The system was calibrated for CIGS films using the Standard–adjusted FP (fundamental parameters) method[2,3] CIGS films of different compositions and thicknesses were measured using XRF. The film thickness was then measured using a stylus profilometer, and the film composition was measured using an inductively-coupled plasma (ICP) spectrophotometer with CIGS standard solutions. The XRF system was calibrated using these values. When these same CIGS layers are

incorporated into standard (Mo/CIGS/CdS/iZnO/AZO) devices, the current-voltage curves yield power conversion efficiencies in the range of 15-18%.

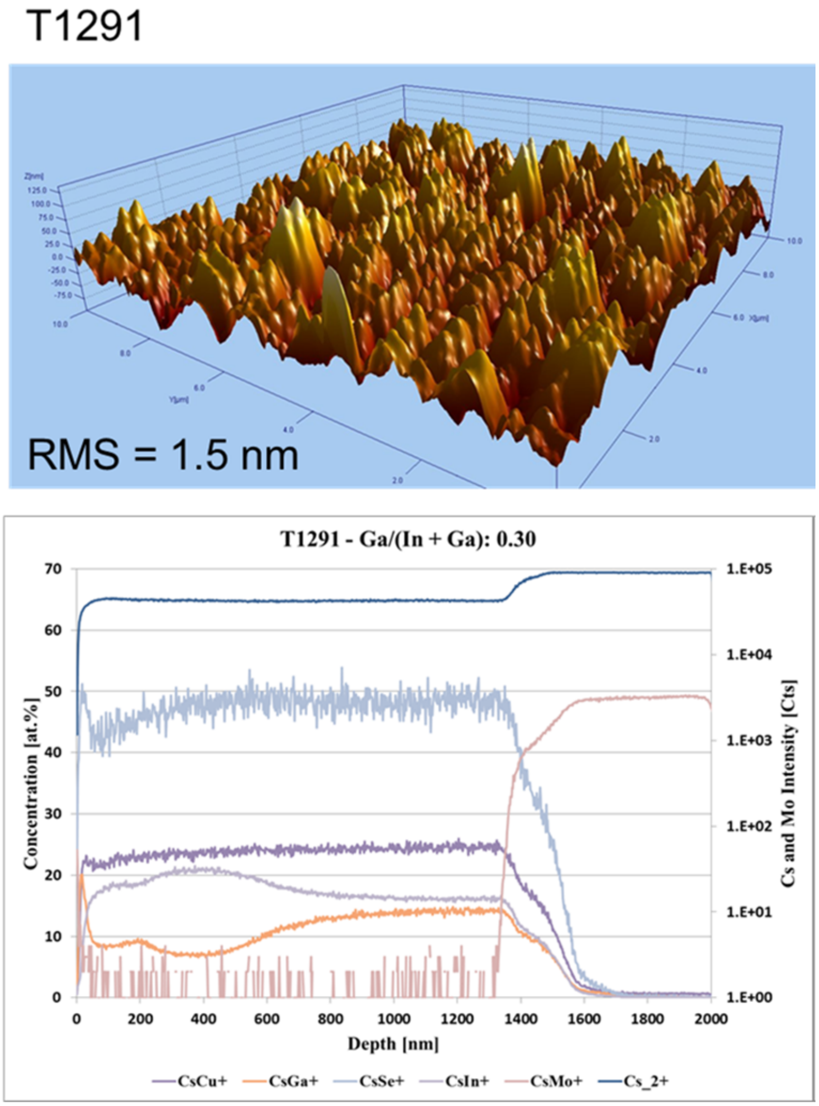

Figure S2. **AFM and TOF-SIMS on CIGS/Mo/SLG substrate.**

2) Device fabrication

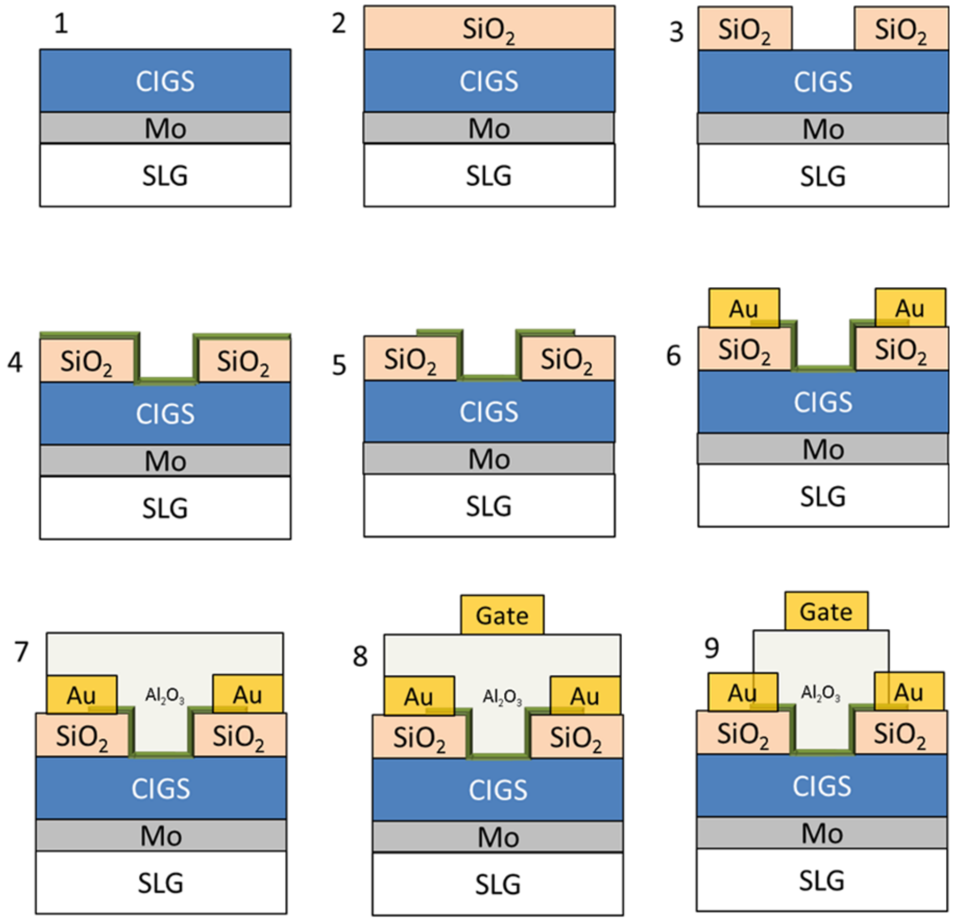

Figure S3. Device fabrication steps for GR/CIGS/Mo/SLG. (1) Clean the CIGS/Mo/SLG via Acetone and IPA. **(2)** Deposit 400 nm of SiO_2 using PECVD. **(3)** Optical/Ebeam lithographically pattern substrates and etch the SiO_2 to expose CIGS devices areas (i.e. 10μm^2, 100μm^2, etc.). **(4)** Wet-transfer CVD graphene (green line) on the opening. **(5)** Optical/Ebeam pattern and etch of the CVD graphene. **(6)** Optical/Ebeam lithographic pattern and deposit Cr (3 nm)/Au (30 nm) contacts on graphene. **(7)** Deposition of 200 nm of Al_2O_3 using ALD. **(8)** Optical/Ebeam lithography of the top-gate contact. **(9)** Optical/EBeam lithography and etch of the Al_2O_3 to expose the Au contacts.

3) Characterization methods

TEM

TEM thin-film samples were made via FIB using a FEI Helios Nanolab 600 Dualbeam (FIB/SEM) system employing a standard in-situ lift-out technique. Initial bulk milling was performed at 30 keV using a liquid metal gallium source. The specimen was subsequently cut free and transferred to a copper grid with a sharpened tungsten Omniprobe needle. Once attached to the TEM grid, the sample was thinned with progressively lower beam voltages down at to an approximate thickness of 50 nm with final milling performed at 2 keV. TEM images were acquired on a JEOL JEM2100F HRTEM operating at an accelerating voltage of 200 keV utilizing a 2K x 2K CCD camera.

EDS

The energy-dispersive X-ray spectroscopy (EDS) chemical maps were acquired on a Hitachi2700C-STEM operated at 200kV.

SIMS

A Physical Electrons 6650 Quadrupole SIMS was used to calculate the concentration of Na with respect to depth in the CIGS and glass samples. A CIGS and Obsidian implanted standard was used to determine the sputter rate and concentration of Na in the samples. The samples were first loaded into the sample exchange chamber and pumped down to 10^{-8} torr before being inserted to the main chamber. Cesium bombardment with a 60 degree angle of incidence, an accelerating voltage of 5 keV, and a beam current of 450 nA was used at 10^{-9} Torr in order to create the ions. The area scanned by the cesium beam had a raster size of 500x500μm and a 10% gate detection area. Charging at the surface was reduced using an electron beam. An electron multiplier detector was used to detect the positive secondary ions.

I-V

Low temperature current-voltage (I-V) measurements were performed using the MMR technologies variable temperature micro-probe system. The temperature is varied from 83-300K in steps of 10K using a micro miniature refrigerator that cools the sample using the Joule-Thompson expansion of high-pressured nitrogen gas (chamber pressure <1mTorr). At each temperature an IV curve is extracted using a Kiethley 2600 source meter.

RAMAN

Raman measurements were made using a WiTec Probe confocal Microscope at 514nm at 100x magnification. The optical power was kept at threshold in order not to locally heat the sample.

4) XPS measurements on SLG SIMS calibration

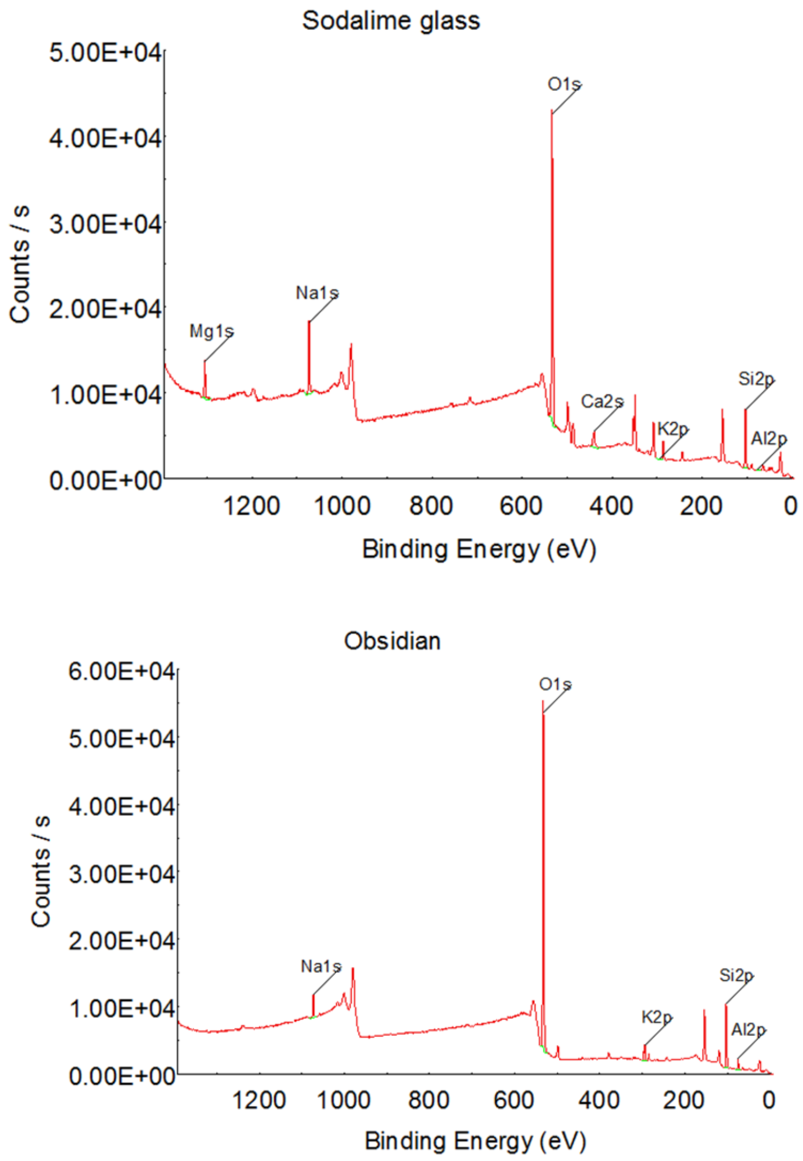

Figure S4. XPS analysis of the top 10 nm of soda-lime glass and calibration sample Obsidian.

Soda-lime glass (after removing ~25nm top layer by Ar+ sputtering)

- Used Obsidian reference to obtain sensitivity factors for all elements except Ca and Mg (XPS was not sensitive enough to detect Ca and Mg).
- Using SiO_2 density the following where measurements were obtained
- 8% wt. of Na_2O (±2.5%)
- $1.15x10^{21}$ Na atoms/cm^3 (~5% atomic Na) for SLG

TOF-SIMS measurements

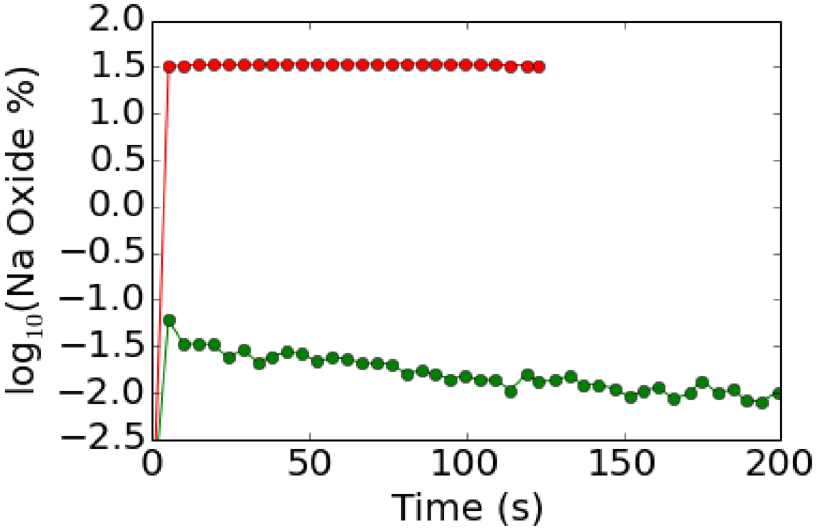

Figure S5. Na depth-profiles in SLG (red) and BSG (green) from (TOF)-SIMS. Data is calibrated using XPS analysis (Fig. 4) giving 1.15x10^{21} and 2.30x10^{20} Na atoms/cm^3 in the SLG and BSG respectively.

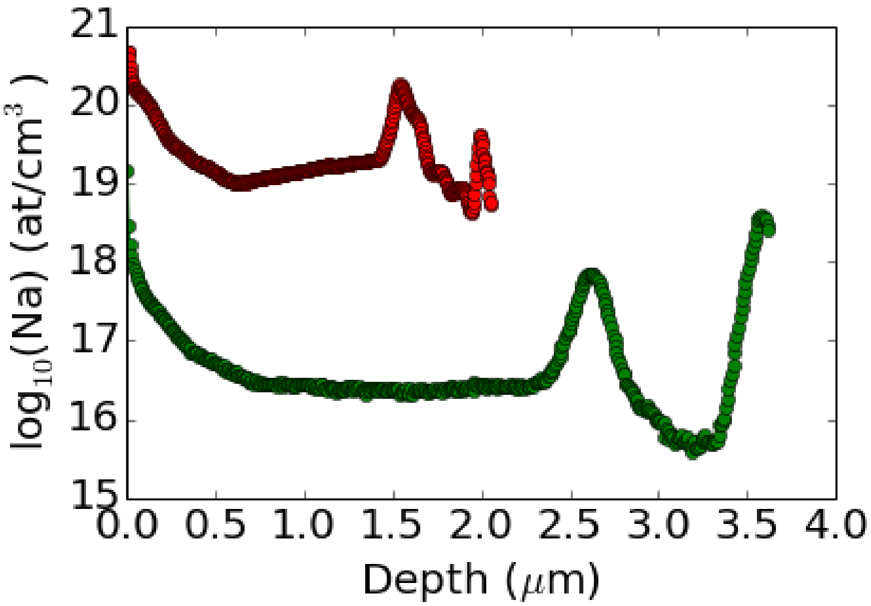

Figure S6. TOF-SIMS measurement on the CIGS/Mo/SLG (Ecoguard Mo-1, 3.0mm thickness and model and Na_2O 8%) (Red) and CIGS/Mo/Borosilicate glass (Green) (Corning 1737, with <1% Na_2O). Na peak at the CIGS-Mo edge at (1.5μm, 2.5μm) in (red, green) curve is from trace Na impurities in the Mo sputter target localized near the Mo-CIGS interface.

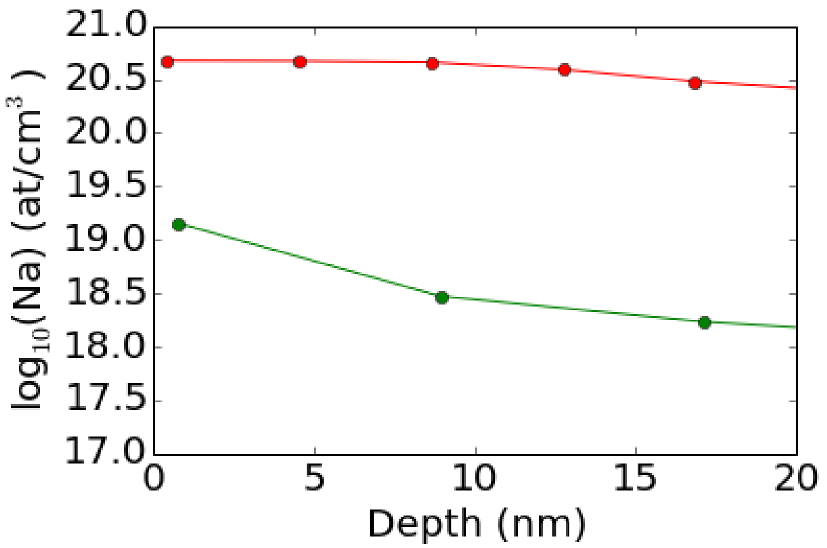

Figure S7. SIMS measurement on the top 20 nm of CIGS/Mo/SLG (Red, 8% Na_2O) (red-curve) and CIGS/Mo/Borosilicate glass (Corning 1737, with <1% Na_2O) (green-curve). SLG surface Na density at 1nm is $4.18x10^{13}cm^{-2}$, BSG surface Na density at 1nm is $6.16x10^{11}$ cm^{-2}.z

5) Transconductance measurements

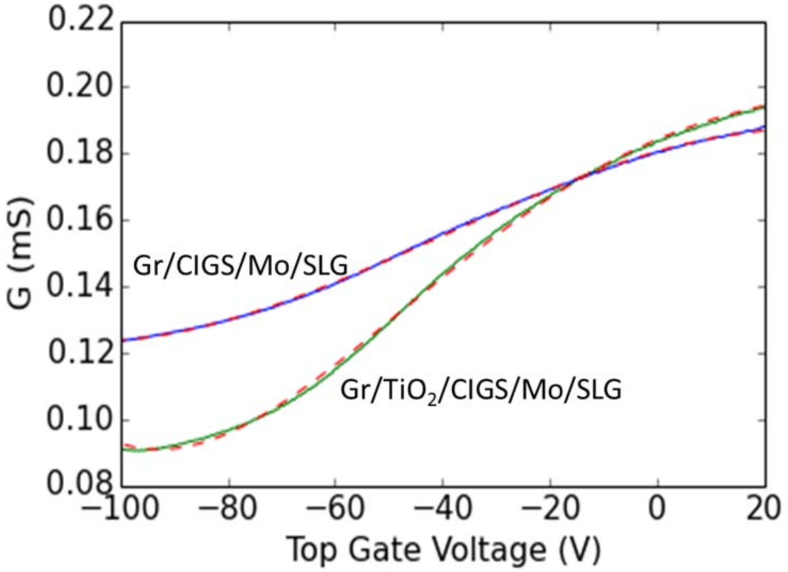

Figure S8. GR/CIGS/Mo/SLG (blue) and GR/TiO_2/CIGS/Mo/SLG (green) transconductance measurements at 10 mV source-drain bias at room-temperature in the dark. Dirac point of GR/CIGS/Mo/SLG is -106V and GR/TiO_2/CIGS/Mo/SLG is -92 V by a polynomial fit to the data. TiO_2 thickness is 4 nm.

The shift in the Fermi Energy (ΔE_F) can be written using Eq. (S1) where $\boldsymbol{v_F}$ is the Fermi velocity ($\boldsymbol{v_F \approx 10^6 m/s}$ for graphene), *N* is the charge density of graphene, and ћ is Planck's constant[4].

$$\Delta E_F = \hbar \boldsymbol{v}_{\mathrm{F}} \sqrt{\pi N} \qquad \text{(S1)}$$

In Eq. (S1) the charge density *N* is calculated considering the capacitance of the top dielectric (C_{TOP}) and the applied potential (*V*) considering a parallel-plate capacitor with ($N = \epsilon_o \epsilon AV/d$) where ϵ_o, ϵ, A, d and V are vacuum permittivity ($\epsilon_o = 8.854 \times 10^{-12}$ F/m), relative dielectric constant ($\epsilon = 9.1$), cross-sectional area ($A = 10^4\ \mu m^2$), and thickness of the dielectric (d = 252nm from TEM measurements).

At the asymptotic top-gate potential at the charge neutrality point (-106 V), *N* and ΔE_F are calculated as 2.11×10^{13} e/cm^{-2} and 536meV, respectively for the GR/CIGS/Mo/SLG substrate. From this calculation, the graphene Fermi level at the charge neutrality point of -106 V is shifted up by ΔE_F = +536meV, with respect to un-doped graphene.

6) Helmholtz equation to calculate the graphene work-function shift on GR/CIGS/SLG (BSG)

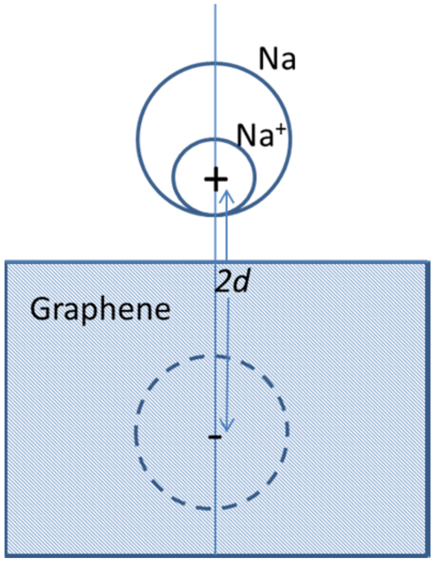

Figure S9. Change of the potential on graphene due to the dipole moment from Na^+ and image-charge.

The change of the potential on graphene due to the dipole moment from Na^+ and image-charge is given by:

$$\Delta\Phi = \frac{2\pi e N_a \mu(\theta)}{\epsilon\epsilon_o},$$

where $\Delta\Phi$ is the change in the Graphene work function, μ is the dipole-moment of the Na^+ and the image force on graphene, ϵ is the relative dielectric constant, ϵ_o is the vacuum permittivity, e is electron charge, N_a is the density of the ions per unit area on CIGS, and θ is the fractional coverage of the ions on graphene.

$$\mu(\theta) = 2dqN_a,$$

where d is the distance between graphene and the Na ion, and q is the charge of an electron. Taking d = 0.1 nm (i.e. the Na^+ radius is 98 pm and elemental Na radius 186 pm), q = 1.6 X 10^{-19} C, N_a = 4.18x10^{13} cm^2 (CIGS/SLG) and 6.16x$10^{11}$$cm^2$ (CIGS/BS), $\epsilon = 13.6$ and $\epsilon_o = 8.854 * 10^{-12}$ F/m, and θ is taken as 1.0 (complete coverage). θ varies from 0.25-1.0 in previous studies[5]. The change in the graphene work function is calculated to be:

- $\Delta\Phi$ for CIGS/SLG = 0.69 eV
- $\Delta\Phi$ for CIGS/BSG = 0.01 eV.

7) GR/CIGS/Mo/SLG photovoltaic device measurements

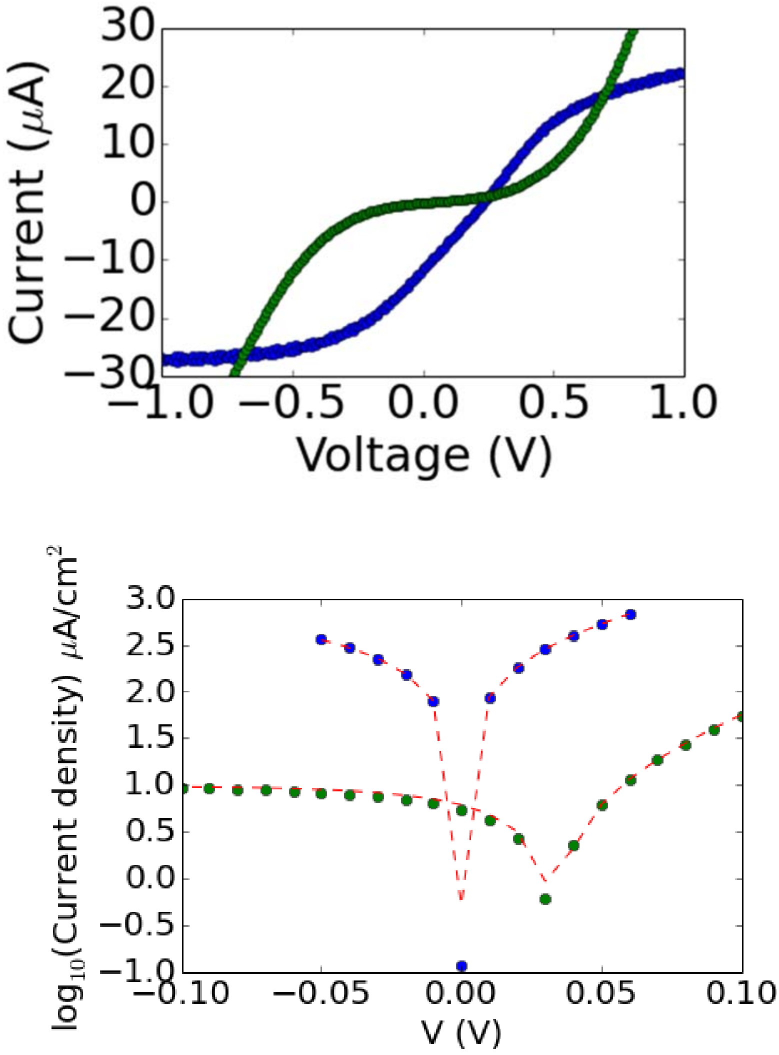

Figure S10. Linear photovoltaic current-voltage (I-V) behavior of the GR/CIGS/Mo/SLG (top). Blue curve is before Al_2O_3 and green curve is after Al_2O_3 under 11.14 mW/cm^2 illumination. After Al_20_3, J_{0} = 3.59x10^{-10} (A/cm^2) and ideality-factor A=1.29. Before Al_20_3: J_0= 1.23x10^{-6} (A/cm^{-2}) and A=91.53 fitting to the low-bias region (bottom).

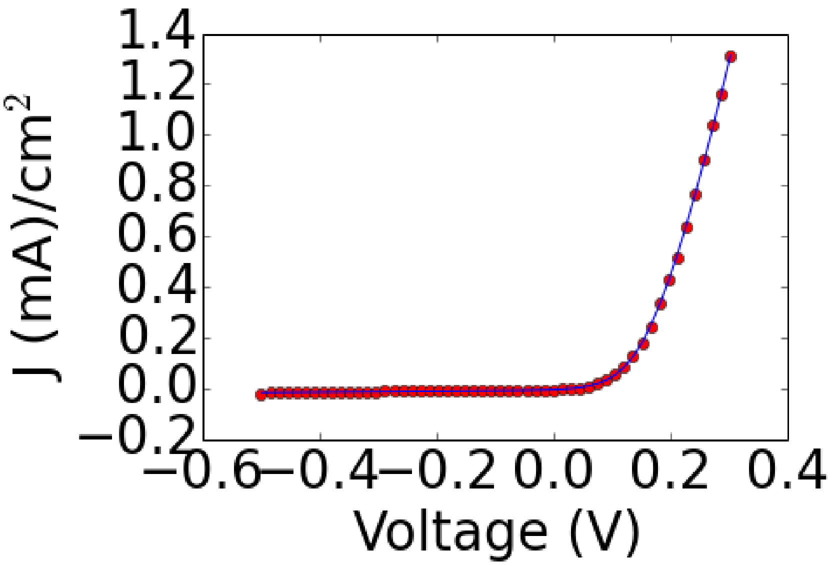

Figure S11. Dark I-V of GR/CIGS/Mo/SLG device with an ideality factor of A=1.21.

8) V_G dependent I-V measurement in GR/CIGS/Mo/SLG

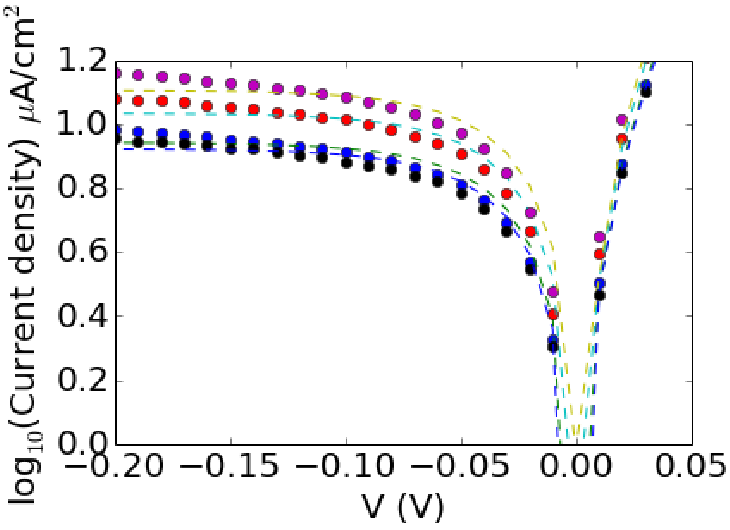

Figure S12. I-V behavior in the dark with different top-gate potentials shown in Table T1.

V_G (V)	J_0 (A/cm^2)	n	Data point color
50	8.2×10^{-10},	1.22	black
0	8.56×10^{-10},	1.21	blue
-50	1.02×10^{-9},	1.22	red
-100	1.17×10^{-9}	1.23	purple

Table ST1. Fitting parameters at different V_G. *n* is the ideality factor, V_G is gate-voltage, and J_0 is the reverse saturation current density.

J_0 vs. V_G relationship at constant T

Graphene/CIGS can be modeled as an n-type metal and p-type semiconductor Schottky diode in which the I-V is behaving as given in Eq. (S2) where *T* is temperature, *k* is the Boltzmann coefficient, *q* is the charge of an electron, *V* is the applied bias, *A* is the contact area and *n* is the ideality factor. J_0 is the reverse saturation current density, which is given in Eq. (S3), where Φ_b is the Schottky barrier height, and A^* is the Richardson coefficient.

$$J = J_o\left[\exp\left(\frac{qV}{nkT}\right) - 1\right] \quad \text{(S2)}$$

$$J_o = A^* T^2 \exp\left(\frac{-\Phi_b}{kT}\right) \quad \text{(S3)}$$

Φ_b is given by Eq. (S4), where φ_G is the Fermi level of graphene and $\boldsymbol{\chi}$ is the ionization potential of CIGS. As given in Eq. (S5), the Fermi level of graphene has an intrinsic component given by $\varphi_{Go}$and a top-gate tunable electrostatic shift of the work function (Δ_F) which is given by Eq. (S6), where *n* is the ideality factor, $\hbar$ is the modified Planck's constant, V_F is the Fermi velocity in graphene, and *N* is the charge density.

$$\Phi_b = \chi - \varphi_G \quad \text{(S4)}$$

$$\varphi_G = \varphi_{Go} - \Delta E_F \quad \text{(S5)}$$

The charge density *N* is a function of V_G as given by Eq. (S7), where ϵ, ϵ_o, A, V_G, *d*, and ΔE_F are the relative dielectric constant, vacuum permittivity, area, gate-bias, thickness of the top dielectric, and the Fermi energy shift due to n-doping, respectively.

$$\Delta E_F = e\Delta V_F = \hbar v_F\sqrt{\pi N} \quad \text{(S6)}$$

$$N = \frac{\epsilon\epsilon_o A(V_G + \Delta V_F)}{d}, \quad \text{(S7)}$$

where ΔV_F represents the offset voltage in the doped graphene due to the Fermi energy shift, *e* is the electron charge (1.6 x 10^{-19} C), $\hbar$ is Planck's constant (4.14 x 10^{-15} eV s), and v_F is the Fermi velocity ($v_F \approx 10^6 m/s$ for graphene). Combining Eqs. (S4-S7) with Eq. (S3) gives an equation between V_G and J_0 given in Eq. (S8). Plotting, ln(J_o) vs. $(|V_G + \Delta V_F|)^{1/2}$ should be a line with an intercept equal to ln(A^*T^2) – ($\boldsymbol{\chi}$-φ_{Go})/kT, as shown in Eq. (S9). Therefore, we are able to obtain the value of the Φ_{bo}, which is the Schottky barrier of the n-doped device at zero-gate bias, independent of the conventional temperature dependent I-V characteristics.

$$J_o = A^* T^2 exp\left(\frac{-(\chi - \varphi_{Go}) - \hbar v_F\sqrt{\frac{\pi\epsilon\epsilon_o A(V_G + \Delta V_F)}{d}}}{kT}\right) \quad \text{(S8)}$$

$$\ln(J_o) = \ln(A^*T^2) + \left(\left\{-\Phi_{bo} - \hbar v_F\sqrt{\frac{\pi\epsilon\epsilon_o A(V_G+\Delta V_F)}{d}}\right\}/kT\right) \tag{S9}$$

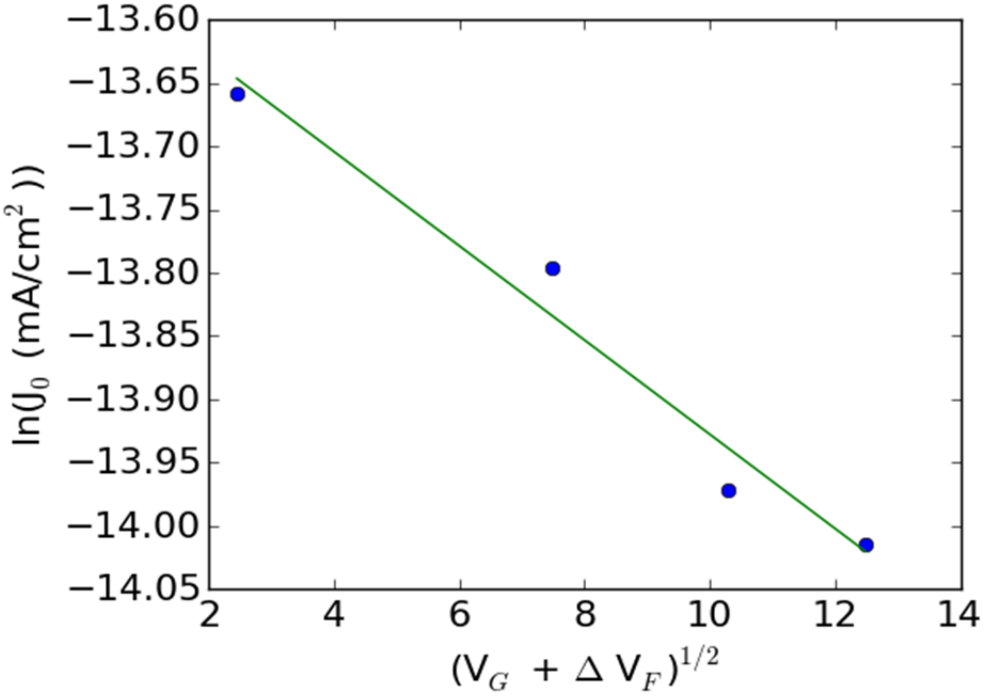

Figure S13. ln(J$_0$) vs. ($|\boldsymbol{V_G} + \boldsymbol{\Delta V_F}|$)$^{1/2}$.

Taking the Richardson constant (A^*) as $A^*=1.18 \times 10^{-6}$ mAcm^{-2}K^{-2} (from the temperature dependent analysis in Fig. S15), the intercept in Fig. S13 at $(V_G + \Delta V_F)$=0 is -13.56:

-13.56= -Φ_{bo}/kT + ln(300 x 300 x 1.18 x 10^{-6}) = -Φ_{bo}/kT + ln(0.106)

Φ_{bo}/kT = 13.56 – 2.24 = 11.31

Φ_{bo}= 11.31 x 26 meV = 0.29 eV

9) Recombination analysis on GR/CIGS/Mo/SLG

For an ideal Schottky barrier, $J_o = A^*T^2\exp\left(\frac{-\Phi_b}{kT}\right)$ where Φ_b is the Schottky barrier height. By fitting $\ln\left(\frac{J_o}{T^2}\right)$ vs. 1000/T, we obtain Schottky barrier height of ϕ_b = 0.11eV and A* = 1.18x10^{-6} $mAcm^{-2}K^{-2}$. This is assuming a fixed Richardson constant A*.

For finite density of states, A* becomes temperature dependent and the Landauer transport model is used as given in Eq. (S10) below[6]:

$$J_o = \left[\frac{qD_o}{\tau}(k_BT)^2\left(\frac{\Phi_B}{k_BT}+1\right)\right]e^{-\Phi_B/k_BT}, \quad \text{(S10)}$$

where $D_0 = [2/(\pi(\hbar\, v_F)^2]$ with $\hbar$ Planck's constant and v_F the Fermi velocity ($v_F \approx 10^6 m/s$ for graphene) represents the prefactor that gives the graphene density of states when multiplied by the energy, and τ is the time scale for carrier injection from the contact. In order to fit this equation to the data shown in the main panel of Fig. 3a, we find the best-fit values for two parameters: $c \equiv \frac{qD_o}{\tau}$ and Φ_B. The blue solid best-fit line shown in the main panel to Fig. 3a is Eq. (S10) with best-fit parameter values of $c \equiv \frac{qD_o}{\tau}$ = 58.15 and $\Phi_B = 0.13\ eV$. We observe that the diode quality factor is temperature dependent which means that there is a strong contribution of tunneling in the recombination mechanism. High ideality factors at low temperatures suggest a transition from tunneling dominated interface recombination at low temperatures to standard Shockley-Read-Hall (SRH) behavior at room temperature.

The same J_0 data can be used in a different model where the diode quality factor, *n*, is taken into account. In this model the reverse saturation current, J_0 can be written as a function of temperature *T*, activation energy, E_a, the diode quality factor, *n*, and J_{00}, which is a weakly temperature-dependent prefactor.

$$J = J_0\exp\left(\frac{qV}{nkT}\right) = J_{00}\exp\left(\frac{-E_a}{nkT}\right)\exp\left(\frac{qV}{nkT}\right), \quad \text{(S11)}$$

Compared to the typical definition of activation energy, where the exponential term containing E_a does not include the ideality factor *n* in the denominator, Eq. (S11) includes *n* in the denominator of both exponential terms to account for the temperature dependence of *n*. In the case of tunneling, where the ideality factor *n* becomes temperature dependent, we can rewrite Eq. (S11) to obtain

$$n\,ln(J_0) = \left(\frac{-E_a}{kT}\right) + n\ln(J_{00}) \ . \quad \text{(S12)}$$

Assuming that J_{00} is temperature independent, we can extract the activation energy E_a using a modified Arrhenius plot (Ref. S16), $n\ ln(J_0)$ vs. $1/T$ (in contrast to a standard Arrhenius plot - $ln(J_0)$ vs. $1/T$ – that one would use when the denominator of the exponential term in Eq. (S11) containing E_a does not include the ideality factor *n*). In an

intrinsic semiconductor, the activation energy is equal to half the bandgap, and for a p-doped semiconductor it should be close to the bandgap energy since the Fermi energy is close to the valence band. We extract an activation energy $E_a = 0.96$ eV which is lower than the CIGS bandgap of 1.15 eV, suggesting a contribution of tunneling enhanced interfacial recombination (Ref. S16). We note that in our case, and in previous studies[17,18], when extracting E_a for Schottky barrier devices, the difference between E_a and Φ_B is largely determined by the value of n and its temperature dependence.

Hole blocking layer between n-graphene and CIGS

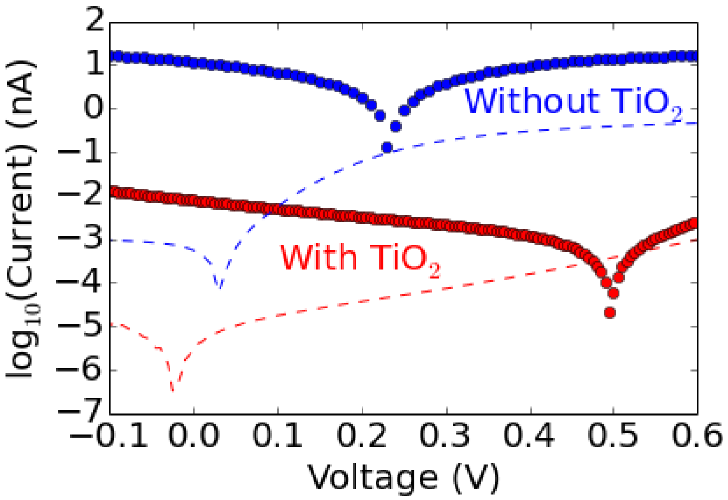

Figure S14. Graphene/CIGS/Mo/SLG (blue) and Graphene/TiO_2/CIGS/Mo/SLG (red) I-V measurements in the dark (dashed) and 11.14 mW white-light (solid). The open circuit voltage without TiO_2 is $V_{OC} = 0.23$ V, and with a 4nm TiO_2 hole blocking layer $V_{OC} = 0.49$ V.

10) Density functional theory (DFT) calculations

DFT calculation of a free standing graphene monolayer

The graphene monolayer crystal structure is described by the primitive translation vectors:

$$\overrightarrow{a_1} = a\left(\frac{\sqrt{3}}{2}, \frac{1}{2}\right)$$

$$\overrightarrow{a_2} = a\left(\frac{\sqrt{3}}{2}, \frac{1}{2}\right)$$

$$\overrightarrow{a_3} = a(0,0,6)$$

where a = 2.46Å is the lattice constant. $\overrightarrow{a_3}$ is the lattice vector in the z direction, which adds a 6Å vacuum gap between graphene layers. Since we use periodic boundary condition in three directions, the vacuum guarantees that the graphene monolayer is isolated from adjacent layers. The atomic positions are:

$$\overrightarrow{p_1}(C) = (0,0)$$

$$\overrightarrow{p_2}(C) = a\left(\frac{1}{\sqrt{3}}, 0\right)$$

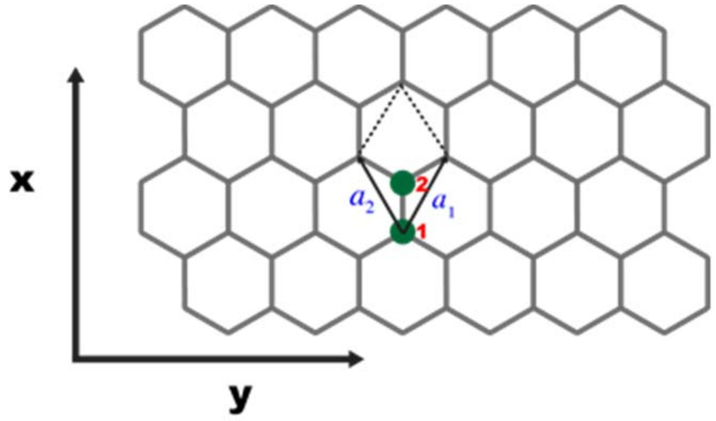

Figure S15. The honeycomb structure of the graphene monolayer. a_1 and a_2 are the two lattice vectors in x,y plane. The primitive unit cell is shown by the lattice vectors and the dotted line and 1 and 2 denote the two carbon atoms per unit cell.

Density functional theory calculation of free-standing graphene with Na

The graphene-monolayer-with -sodium crystal structure is described by the primitive translation vector as shown in:

$$\overrightarrow{a_1} = a\left(\frac{\sqrt{3}}{2}, \frac{1}{2}, 0\right)$$

$$\overrightarrow{a_2} = a\left(\frac{\sqrt{3}}{2}, \frac{1}{2}, 0\right)$$

$$\overrightarrow{a_3} = a(0,0,12)$$

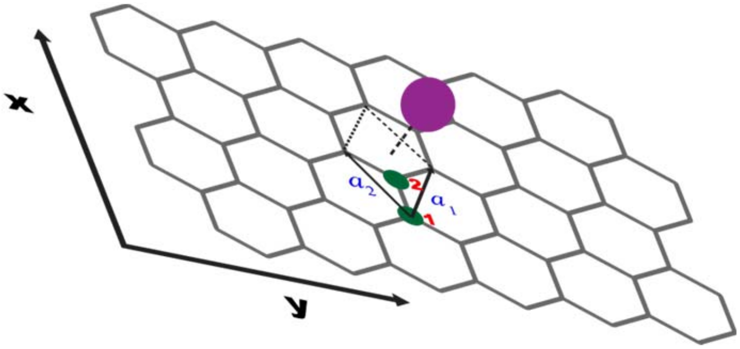

Figure S16. The honeycomb structure of the graphene monolayer with sodium (purple). a_1 and a_2 are the two lattice vectors in x,y plane. The primitive unit cell is shown by the lattice vectors and the dotted line and 1 and 2 denote the two carbon atoms per unit cell; the purple atom is the sodium atom in the primitive unit cell.

Since we introduce the sodium atom into the graphene monolayer crystal structure, we add a thicker vacuum layer (12Å) to isolate the graphene-monolayer-with-sodium system. The atomic positions are:

$$\overrightarrow{p_1}(C) = (0,0,0)$$

$$\overrightarrow{p_2}(C) = a\left(\frac{1}{\sqrt{3}}, 0,0\right)$$

$$\overrightarrow{p_3}(Na) = a\left(\frac{1}{\sqrt{3}}, 0,1\right)$$

All the calculations are performed by DFT, which is implemented in GPAW based on the projector-augmented wave (PAW) method and the atomic simulation environment (ASE) (Ref. S7-S9). We use the default Finite Difference mode, which means that the wave function will be expanded on a real space grid, and the grid spacing is 0.18Å. The Perdew-Burke-Ernzerhof (PBE), which is a generalized gradient approximation (GGA)

type, is adopted. The Brillouin-zone is sampled using Γ-centered 5 × 5 × 1 Monkhorst-Pack k-points. The electrons will be distributed into the available energy levels according to the Fermi Dirac Distribution, and the width kB T = 0.05 eV. The convergence criterion is 1e-12 for the Poisson solver.

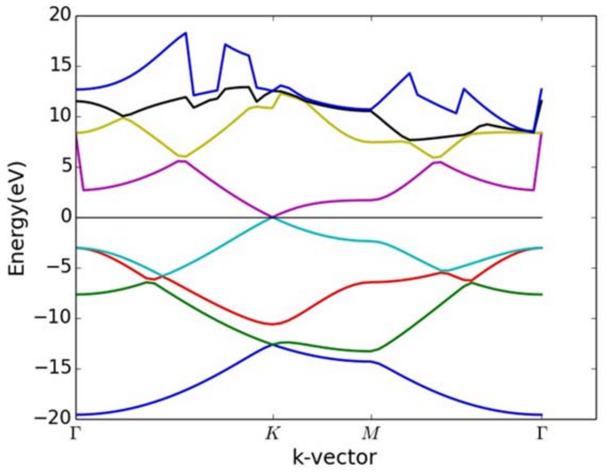

Figure S17. DFT calculation of the band-structure of the graphene monolayer. It clearly shows the linear behavior of the bands near the vicinity of K at the Fermi energy. The conduction and valence bands cross at the Dirac point indicating no intrinsic doping of the structure.

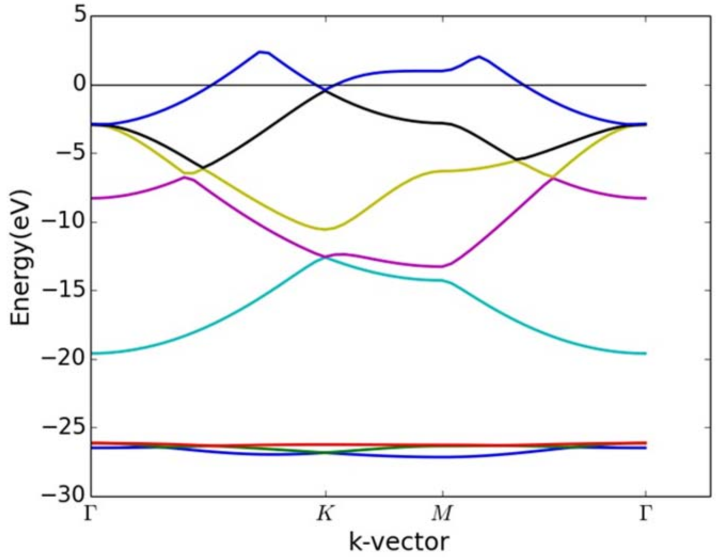

Figure S18. DFT calculation of the band- structure of graphene monolayer interacting with the Na (density: 1 Na atom per primitive unit cell). The result shows a 474meV shift in the Fermi level from the Dirac point at K, which indicates n-doping of graphene from Na.

11) C-V measurements

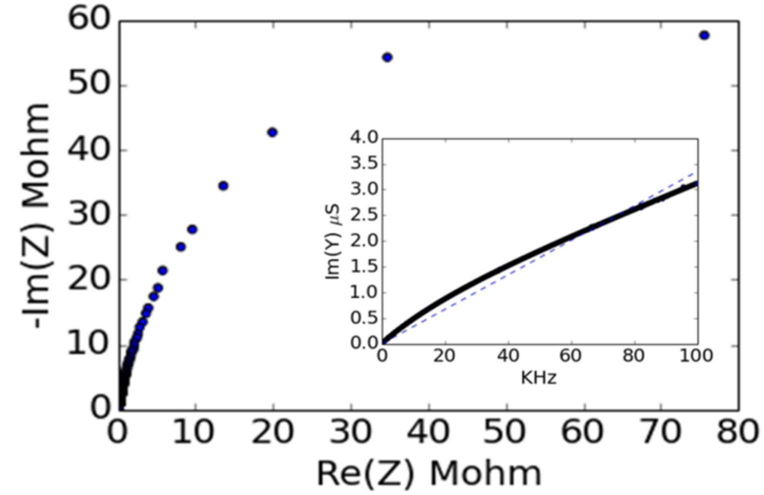

Figure S19. C-V measurement on GR/CIGS/Mo/SLG at 50 mV_{rms} a.c. voltage at 0 V d.c. bias in the frequency range 100 Hz – 100 KHz in dark.

Considering the junction as an RC-equivalent circuit, the junction capacitance *(C_p)* is calculated as 53.32 nF/cm^2 by plotting $Im\left(\frac{1}{Z}\right)$ vs. f (inset) using Eq. (S13). Considering uniform charge density in the space-charge region, the space-charge width (W_d) is calculated to be 190.94 nm.

$$Im\left(\frac{1}{Z}\right) = 2\pi jCf \qquad \text{(S13)}$$

References

[S1] Al-Thani, H. & Williamson, D. The Effect of Mo Back Contact on Na Out-Diffusion and Device Performance of Mo/Cu, *NREL/CP-520-32254,* (2002).
[S2] Dwyer, D., Schujman, S., Novak, J., Metacarpa, D. and Haldar, P. Selenium flux effects on Cu(In,Ga)Se2 growth rate, and control by in-line X-ray fluorescence, *Proc. of the 39rd IEEE Photovoltaic Specialists Conference* 1957-1960 (2013).
[S3] Gabor, A. *et al*. Band-gap engineering in Cu(In,Ga)Se_2 thin films grown from $(In,Ga)_2Se_3$ precursors, *Solar Energy Materials and Solar Cells*. **41/42** 247-260 (1996).
[S4] Das, A. *et al*. Monitoring dopants by Raman scattering in an electrochemically top-gated graphene transistor. *Nature. Nanotec.* **3**, (4) 210–215 (2008).
[S5] Caragiu, M. & Finberg, S. Alkali metal adsorption on graphite: a review. *J. of Physics-condensed matter*. **17**, R995–R1024 (2005).
[S6] Sinha, D. & Lee, J. U. Ideal Graphene/Silicon Schottky Junction Diodes. *Nano Lett.* **14**, 4660–4664 (2014).
[S7] Mortensen, J., Hansen, L. & Wedel, K. Real-space grid implementation of the projector augmented wave method. *Phys. Rev. B*. **71**, 035109, (2005).
[S8] Enkovaara, J. *et al*. Electronic structure calculations with gpaw: a real-space implementation of the projector augmented-wave method. *J. of Phys.: Condensed Matter*. **22**, 253202 (2010).
[S9] Bahn, S. & Jacobsen, K. An object-oriented scripting inter- face to a legacy electronic structure code. *Computing in Science & Engineering*. **4**, 56–66 (2002).
[S10] Dong, X. *et al*. Doping Single-Layer Graphene with Aromatic Molecules. *Small* **5,** 1422 - 1426 (2009).
[S11] Wang, Z. *et al*. Air-stable *n*-type doping of graphene from overlying Si_3N_4 film. *Appl. Surf. Sci.* **307,** 712-715 (2014).
[S12] Kim, Y., Yoo, J.M., Jeon, H.R. & Hong, B.H. Efficient n-doping of graphene films by APPE (aminophenyl propargyl ether): a substituent effect. *Phys. Chem. Chem. Phys.* **15,** 18353-18356 (2013).
[S13] Park, J. *et al*. Work-Function Engineering of Graphene Electrodes by Self-Assembled Monolayers for High-Performance Organic Field-Effect Transistors. *J. Phys. Chem. Lett.* **2,** 841–845 (2011).
[S14] Zhang, C. *et al*. Synthesis of Nitrogen-Doped Graphene Using Embedded Carbon and Nitrogen Sources. *Adv. Mater.* **23,** 1020-1024 (2011).
[S15] Huh, S. *et al*. Selective n-Type Doping of Graphene by Photo-patterned Gold Nanoparticles. *ACS Nano* **5,** 3639–3644 (2011).
[S16] Nadenau, V., Rau, U., Jasenek, A. & Schock, H.W. Electronic properties of CuGaSe2-based heterojunction solar cells. Part I. Transport analysis. *J. Appl. Phys.* **87**, 584 - 593 (2000).
[S17] Asghar, M., Mahmood, K., Faisal, M., and Hasan, M. A. Electrical characterization of Au/ZnO/Si Schottky contact. *J. Phys.: Conf. Ser.* **439,** 012030 (2013).
[S18] Wu, C. S. *et al.* Novel GaAs/AlGaAs Multiquantum-Well Schottky-Junction Device and its Photovoltaic LWIR Detection. *IEEE Transactions on Electron Devices* **39**, 234-241 (1992).
